# Supplementary material for: Effectiveness and Safety of Immunosuppressants and Biological Therapy for Chronic Spontaneous Urticaria: A Network Meta-Analysis
Source: Biomedicines. 2022 Sep 1;10(9):2152. doi: 10.3390/biomedicines10092152 (PMC9496015; doi:10.3390/biomedicines10092152)
Supplement: Supplementary file 1 [file biomedicines-10-02152-s001.zip › biomedicines-1853844-supplementary.pdf]

Table S1. Study inclusion and exclusion criteria

Category	Inclusion criteria	Exclusion criteria
Population	Symptomatic CSU patients (12 years or older) despite being under antihistamine (standard dose or up to a 4-fold dose) treatment	1. Studies that recruited participants aged less than 12 years or unclear definition of CSU
		2. Non-human study
Interventions	Selected pharmacological treatment for symptomatic CSU patients despite being under antihistamine (standard dose or a 4-fold dose) treatment, including immunosuppressants such as methotrexate, cyclosporin, hydroxychloroquine, azathioprine, biological therapy, omalizumab, and the leukotriene receptor antagonist, montelukast	1. Studies without pharmacological treatment
		2. Studies that used other medications which did not fit our inclusion criteria
Comparators	Placebo plus antihistamine (standard dose or a 4-fold dose) or active comparators plus antihistamine (standard dose or a 4-fold dose)	Studies without a control group
Outcomes	Effectiveness evaluation:<br>weekly urticaria activity score (UAS7), Dermatology Life Quality Index (DLQI) .<br>Safety evaluation:<br>Patients experienced at least one adverse event.	1. Studies not providing suitable evaluation scores
		2. Studies not providing enough information regarding the effectiveness evaluation
Study type	Randomized control trial (RCT)	Non-RCTs, including case reports/case series, systematic reviews, and meta-analyses
Publication timing	Studies recruited from Jan. 2000 to 31 July 2021	Studies not published in the recruitment window

CSU, chronic spontaneous urticaria.

Table S2. Characteristics of excluded studies

Source	Study design (no. of patients)	Age, mean (SD), yr	Gender	Comparator
Bhanja et al.[31] 2015	SB, RCT (*N*=52)	≥ 12	N/A	AZA<br><br>Placebo
Pathania et al.[32] 2019	Randomized prospective active controlled non-inferiority study (*N*=80)	CsA: 35.9 (9.4)<br><br>AZA: 36.7 (9.7)	Female: 60%	AZA<br><br>CsA
Loria et al.[33] 2001	RCT (*N*=20)	CsA: 45 (range: 19~64)<br><br>Pred: 43 (range: 32~57)	CsA:<br>Female: 60%<br><br>Pred:<br>Female: 80%	CsA<br><br>Pred
Gioacchino et al.[34] 2003	RCT (*N*=40)	36.3 (range:18~60)	Female: 70%	CsA: 5 mg/kg/day (8 wks) then 4 mg/kg/day (8 wks)<br><br>Cetirizine 10 mg (2 wks) then CsA: 5 mg/kg/day (8 wks) then 4 mg/kg/day (8 wks)
Vena et al.[35] 2006	RCT (*N*=99)	CsA (16 wks): 44 (9.8)<br>CsA (8 wks): 37.1 (11.3)<br>Placebo (16 wks): 41.7 (11.5)	CsA (16 wks): 54.8%<br>CsA (8 wks): 51.5%<br>Placebo (16 wks): 65.7%	CsA<br>Placebo
Lorenzo et al.[36] 2004	RCT (*N*=160)	Desloratadine: 44 (range: 18~63); Montelukast: 44.4 (range: 18~64); Desloratadine + Montelukast: 43.7 (range: 20~69)<br>Placebo: 43.6 (range: 20~65)	Desloratadine: 67.5%<br>Montelukast: 70%<br>Desloratadine + Montelukast: 67.5%<br>Placebo: 72.5%	Desloratadine<br>Montelukast<br>Placebo
Nettis et al.[37] 2004	RCT (*N*=81)	37.5 (12.3)	Desloratadine: 76%<br>Desloratadine + Montelukast: 84.6%<br>Placebo: 60%	Desloratadine<br>Montelukast<br>Placebo

Godse et al.[38]<br>2006	Randomized, uncontrolled study<br>(*N*=20)	31.2	Female: 60%	Montelukast 10 mg/day<br>Cetirizine 10 mg/day
Wan et al.[39]<br>2009	SB, Randomized study<br>(*N*=120)	Hydroxyzine + Cetirizine: 31 (range: 18~45)<br>Hydroxyzine + Famotidine: 36.4 (range: 20~52)<br>Hydroxyzine + Montelukast: 34.8 (range: 20~54)<br>Placebo: 33.2 (range: 18~48)	Hydroxyzine + Cetirizine: 60%<br>Hydroxyzine + Famotidine: 66.7%<br>Hydroxyzine + Montelukast: 63.3%<br>Placebo: 56.7%	Hydroxyzine<br>Cetirizine<br>Famotidine<br>Montelukast<br>Placebo
Wan et al.[40]<br>2014	SB, Randomized study<br>(*N*=50)	Cetirizine + Famotidine + Montelukast: 34.8 (range: 20~54) Cetirizine + Cetirizine + Famotidine: 36.4 (range: 20~52)<br>Cetirizine + Cetirizine + Montelukast: 22.5 (range: 12~28)	Cetirizine + Famotidine + Montelukast: 56.7%<br>Cetirizine + Cetirizine + Famotidine: 60%<br>Cetirizine + Cetirizine + Montelukast: 70%	Cetirizine<br>Famotidine<br>Montelukast<br>Placebo
Krawczyk et al.[41]<br>2020	Open, randomized trial<br>(*N*=17)	44	Female: 94.1%	Montelukast 10 mg/day<br><br>Placebo
Staubach et al.[42]<br>2018	RCT<br>(*N*=91)	42.9 (12.3)	Intervention:<br>OMA (300): 68.2%<br><br>Placebo:<br>70.2%	OMA (300)<br>Placebo
Metz et al.[43]<br>2019	RCT<br>(*N*=40)	Intervention:<br>OMA (300): 37.5 (11)<br>Placebo: 41.1 (8)	Intervention:<br>OMA (300): 90%<br>Placebo: 80%	OMA (300)<br>Placebo

AZA, azathioprine; CsA, cyclosporin; N/A, not applicable; OMA(300), omalizumab 300 mg/month; Pred, prednisolone; RCT, randomized controlled trial; SB, single-blind; SD, standard deviation; wks, weeks.

Table S3. Summary of risk of bias assessment

Study	Medication	Outcome	Bias arising from the randomization process	Bias due to deviation from the intended intervention	Bias due to missing outcome data	Bias in the measurement of the outcome	Bias in selection of reported results	Overall risk of bias
Grattan et al.[17] 2000	Cyclosporin	UAS7, AE	Low risk	Low risk	Low risk	Low risk	Low risk	Low risk
Sharma et al.[18] 2014	MTX	UAS7, AE	Low risk	Low risk	Low risk	Low risk	Low risk	Low risk
Erbagci et al.[20] 2002	Montelukast	UAS7, AE	Some concern	Low risk	Low risk	Low risk	Low risk	Some concern
Sarkar et al.[21] 2017	Montelukast	UAS7	Low risk	Low risk	Low risk	Low risk	Low risk	Low risk
Maurer et al.[22] 2011	Omalizumab	UAS7, AE	Low risk	Low risk	Low risk	Low risk	Low risk	Low risk
Saini et al.[23] 2011	Omalizumab	UAS7	Low risk	Low risk	Low risk	Low risk	Low risk	Low risk
Maurer et al.[24] 2013 (ASTERIA 2)	Omalizumab	UAS7, DLQI, AE	Low risk	Low risk	Low risk	Low risk	Low risk	Low risk
Kaplan et al.[25] 2013 (GLACIAL)	Omalizumab	UAS7, DLQI, AE	Low risk	Low risk	Low risk	Low risk	Low risk	Low risk
Saini et al.[26] 2015 (ASTERIA 1)	Omalizumab	UAS7, DLQI, AE	Low risk	Low risk	Low risk	Low risk	Low risk	Low risk
Staubach et al.[27] 2016	Omalizumab	UAS7, DLQI	Low risk	Low risk	Low risk	Low risk	Low risk	Low risk
Hide et al.[28] 2017 (POLARIS)	Omalizumab	UAS7, DLQI, AE	Low risk	Low risk	Low risk	Low risk	Low risk	Low risk
Metz et al.[29] 2017	Omalizumab	UAS7	Low risk	Low risk	Low risk	Low risk	Low risk	Low risk

Leducq et al.[19] 2020	MTX	DLQI	Low risk	Low risk	Low risk	Low risk	Low risk	Low risk
Boonpiyathad et al.[30] 2017	HCQ	DLQI, AE	Some concern	Some concern	Low risk	Some concern	Some concern	Some concern

AE, adverse event; DLQI, Dermatology Life Quality Index; HCQ, hydroxychloroquine; MTX, methotrexate; UAS7, weekly urticaria activity score.

(A)

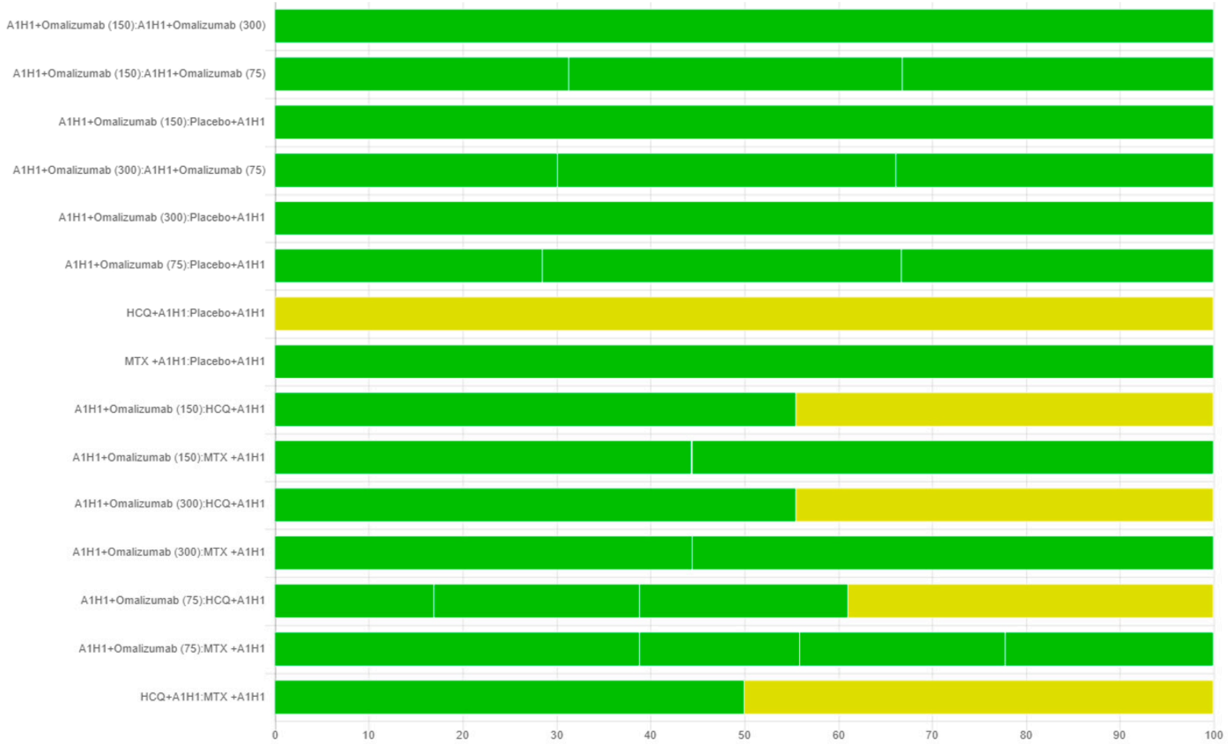
A1H1+Omalizumab (150):A1H1+Omalizumab (300)
A1H1+Omalizumab (150):A1H1+Omalizumab (75)
A1H1+Omalizumab (150):Placebo+A1H1
A1H1+Omalizumab (300):A1H1+Omalizumab (75)
A1H1+Omalizumab (300):Placebo+A1H1
A1H1+Omalizumab (75):Placebo+A1H1
HCQ+A1H1:Placebo+A1H1
MTX +A1H1:Placebo+A1H1
A1H1+Omalizumab (150):HCQ+A1H1
A1H1+Omalizumab (150):MTX +A1H1
A1H1+Omalizumab (300):HCQ+A1H1
A1H1+Omalizumab (300):MTX +A1H1
A1H1+Omalizumab (75):HCQ+A1H1
A1H1+Omalizumab (75):MTX +A1H1
HCQ+A1H1:MTX +A1H1
0
10
20
30
40
50
60
70
80
90
100

(B)

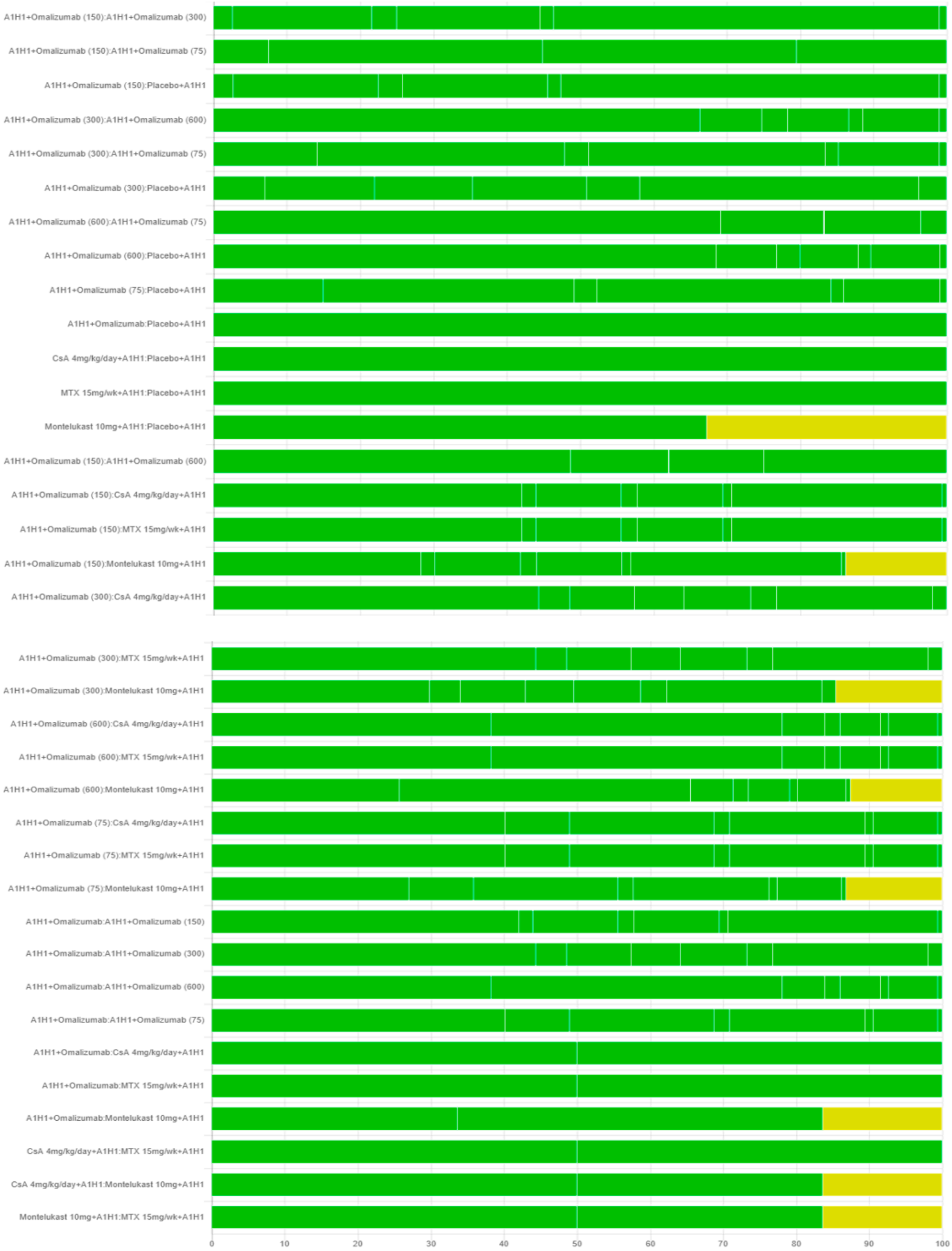
A1H1+Omalizumab (150):A1H1+Omalizumab (300)
A1H1+Omalizumab (150):A1H1+Omalizumab (75)
A1H1+Omalizumab (150):Placebo+A1H1
A1H1+Omalizumab (300):A1H1+Omalizumab (600)
A1H1+Omalizumab (300):A1H1+Omalizumab (75)
A1H1+Omalizumab (300):Placebo+A1H1
A1H1+Omalizumab (600):A1H1+Omalizumab (75)
A1H1+Omalizumab (600):Placebo+A1H1
A1H1+Omalizumab (75):Placebo+A1H1
A1H1+Omalizumab:Placebo+A1H1
CsA 4mg/kg/day+A1H1:Placebo+A1H1
MTX 15mg/wk+A1H1:Placebo+A1H1
Montelukast 10mg+A1H1:Placebo+A1H1
A1H1+Omalizumab (150):A1H1+Omalizumab (600)
A1H1+Omalizumab (150):CsA 4mg/kg/day+A1H1
A1H1+Omalizumab (150):MTX 15mg/wk+A1H1
A1H1+Omalizumab (150):Montelukast 10mg+A1H1
A1H1+Omalizumab (300):CsA 4mg/kg/day+A1H1
A1H1+Omalizumab (300):MTX 15mg/wk+A1H1
A1H1+Omalizumab (300):Montelukast 10mg+A1H1
A1H1+Omalizumab (600):CsA 4mg/kg/day+A1H1
A1H1+Omalizumab (600):MTX 15mg/wk+A1H1
A1H1+Omalizumab (600):Montelukast 10mg+A1H1
A1H1+Omalizumab (75):CsA 4mg/kg/day+A1H1
A1H1+Omalizumab (75):MTX 15mg/wk+A1H1
A1H1+Omalizumab (75):Montelukast 10mg+A1H1
A1H1+Omalizumab:A1H1+Omalizumab (150)
A1H1+Omalizumab:A1H1+Omalizumab (300)
A1H1+Omalizumab:A1H1+Omalizumab (600)
A1H1+Omalizumab:A1H1+Omalizumab (75)
A1H1+Omalizumab:CsA 4mg/kg/day+A1H1
A1H1+Omalizumab:MTX 15mg/wk+A1H1
A1H1+Omalizumab:Montelukast 10mg+A1H1
CsA 4mg/kg/day+A1H1:MTX 15mg/wk+A1H1
CsA 4mg/kg/day+A1H1:Montelukast 10mg+A1H1
Montelukast 10mg+A1H1:MTX 15mg/wk+A1H1
0
10
20
30
40
50
60
70
80
90
100

(C)

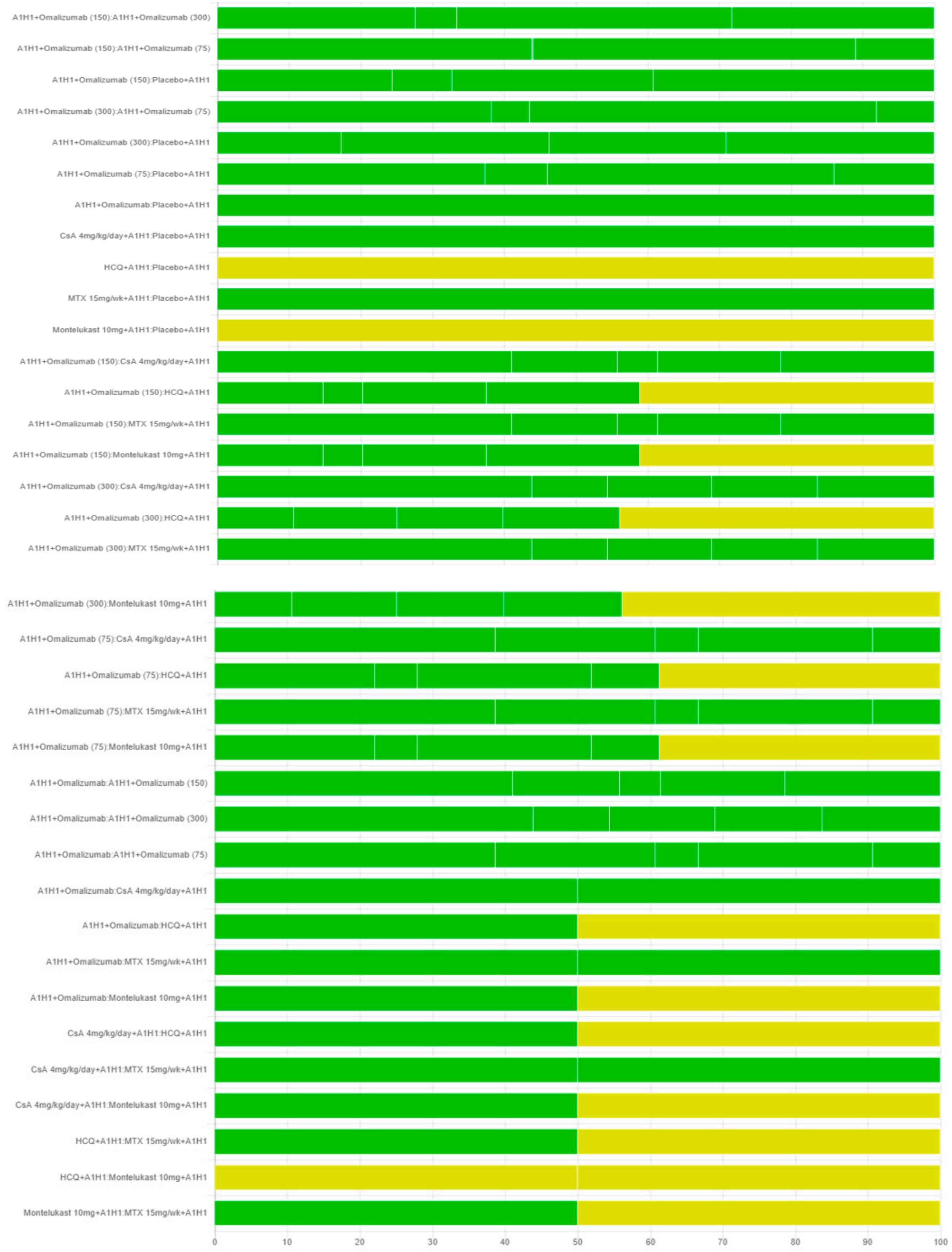

A1H1+Omalizumab (150):A1H1+Omalizumab (300)
A1H1+Omalizumab (150):A1H1+Omalizumab (75)
A1H1+Omalizumab (150):Placebo+A1H1
A1H1+Omalizumab (300):A1H1+Omalizumab (75)
A1H1+Omalizumab (300):Placebo+A1H1
A1H1+Omalizumab (75):Placebo+A1H1
A1H1+Omalizumab:Placebo+A1H1
CsA 4mg/kg/day+A1H1:Placebo+A1H1
HCQ+A1H1:Placebo+A1H1
MTX 15mg/wk+A1H1:Placebo+A1H1
Montelukast 10mg+A1H1:Placebo+A1H1
A1H1+Omalizumab (150):CsA 4mg/kg/day+A1H1
A1H1+Omalizumab (150):HCQ+A1H1
A1H1+Omalizumab (150):MTX 15mg/wk+A1H1
A1H1+Omalizumab (150):Montelukast 10mg+A1H1
A1H1+Omalizumab (300):CsA 4mg/kg/day+A1H1
A1H1+Omalizumab (300):HCQ+A1H1
A1H1+Omalizumab (300):MTX 15mg/wk+A1H1
A1H1+Omalizumab (300):Montelukast 10mg+A1H1
A1H1+Omalizumab (75):CsA 4mg/kg/day+A1H1
A1H1+Omalizumab (75):HCQ+A1H1
A1H1+Omalizumab (75):MTX 15mg/wk+A1H1
A1H1+Omalizumab (75):Montelukast 10mg+A1H1
A1H1+Omalizumab:A1H1+Omalizumab (150)
A1H1+Omalizumab:A1H1+Omalizumab (300)
A1H1+Omalizumab:A1H1+Omalizumab (75)
A1H1+Omalizumab:CsA 4mg/kg/day+A1H1
A1H1+Omalizumab:HCQ+A1H1
A1H1+Omalizumab:MTX 15mg/wk+A1H1
A1H1+Omalizumab:Montelukast 10mg+A1H1
CsA 4mg/kg/day+A1H1:HCQ+A1H1
CsA 4mg/kg/day+A1H1:MTX 15mg/wk+A1H1
CsA 4mg/kg/day+A1H1:Montelukast 10mg+A1H1
HCQ+A1H1:MTX 15mg/wk+A1H1
HCQ+A1H1:Montelukast 10mg+A1H1
Montelukast 10mg+A1H1:MTX 15mg/wk+A1H1
0
10
20
30
40
50
60
70
80
90
100

Figure S1. Risk of bias of bar chart of direct and indirect comparisons between omalizumab and other immunosuppressants. A. Network meta-analysis (NMA) of the Daily Life Quality Index (DLQI) assessment, B. NMA of the weekly urticaria activity score (UAS7) assessment, C. NMA of adverse events assessment. The bar chart shows the contributions of each piece of the study to the network estimate. Vertical lines separate the percentage contributions of different studies. Each bar shows the percentage contribution from studies judged to be at low (green), moderate (yellow), and high (red) risk of bias. We did not have high risk of bias in this network. A1H1, H1-antihistamine; CsA, cyclosporin; HCQ, hydroxychloroquine; MTX, methotrexate.

(A)

Comparison	Number of Studies	Within-study bias	Reporting bias	Indirectness	Imprecision	Heterogeneity	Incoherence	Confidence rating
Mixed evidence								
A1H1+Omalizumab (150) vs A1H1+Omalizumab (300)	3	No concerns	Low risk	No concerns	No concerns	No concerns	No concerns	High
A1H1+Omalizumab (150) vs A1H1+Omalizumab (75)	2	No concerns	Low risk	No concerns	No concerns	No concerns	No concerns	High
A1H1+Omalizumab (150) vs Placebo+A1H1	3	No concerns	Low risk	No concerns	No concerns	No concerns	No concerns	High
A1H1+Omalizumab (300) vs A1H1+Omalizumab (75)	2	No concerns	Low risk	No concerns	No concerns	No concerns	No concerns	High
A1H1+Omalizumab (300) vs Placebo+A1H1	5	No concerns	Low risk	No concerns	No concerns	No concerns	No concerns	High
A1H1+Omalizumab (75) vs Placebo+A1H1	2	No concerns	Low risk	No concerns	No concerns	No concerns	No concerns	High
HCQ+A1H1 vs Placebo+A1H1	1	Some concerns	Low risk	No concerns	No concerns	No concerns	No concerns	Moderate
MTX +A1H1 vs Placebo+A1H1	1	No concerns	Low risk	No concerns	No concerns	No concerns	No concerns	High
Indirect evidence								
A1H1+Omalizumab (150) vs HCQ+A1H1	--	No concerns	Low risk	No concerns	No concerns	No concerns	No concerns	High
A1H1+Omalizumab (150) vs MTX +A1H1	--	No concerns	Low risk	No concerns	No concerns	No concerns	No concerns	High
A1H1+Omalizumab (300) vs HCQ+A1H1	--	No concerns	Low risk	No concerns	No concerns	No concerns	No concerns	High
A1H1+Omalizumab (300) vs MTX +A1H1	--	No concerns	Low risk	No concerns	No concerns	No concerns	No concerns	High
A1H1+Omalizumab (75) vs HCQ+A1H1	--	No concerns	Low risk	No concerns	No concerns	No concerns	No concerns	High
A1H1+Omalizumab (75) vs MTX +A1H1	--	No concerns	Low risk	No concerns	No concerns	No concerns	No concerns	High
HCQ+A1H1 vs MTX +A1H1	--	Some concerns	Low risk	No concerns	No concerns	No concerns	No concerns	Moderate

(B)

Comparison	Number of Studies	Within-study bias	Reporting bias	Indirectness	Imprecision	Heterogeneity	Incoherence	Confidence rating
				Mixed evidence				
A1H1+Omalizumab vs Placebo+A1H1	1	No concerns	Low risk	No concerns	No concerns	No concerns	No concerns	High
A1H1+Omalizumab (150) vs A1H1+Omalizumab (300)	3	No concerns	Low risk	No concerns	No concerns	No concerns	No concerns	High
A1H1+Omalizumab (150) vs A1H1+Omalizumab (75)	2	No concerns	Low risk	No concerns	No concerns	No concerns	No concerns	High
A1H1+Omalizumab (150) vs Placebo+A1H1	3	No concerns	Low risk	No concerns	No concerns	No concerns	No concerns	High
A1H1+Omalizumab (300) vs A1H1+Omalizumab (600)	1	No concerns	Low risk	No concerns	No concerns	No concerns	No concerns	High
A1H1+Omalizumab (300) vs A1H1+Omalizumab (75)	3	No concerns	Low risk	No concerns	No concerns	No concerns	No concerns	High
A1H1+Omalizumab (300) vs Placebo+A1H1	7	No concerns	Low risk	No concerns	No concerns	Some concerns	No concerns	Moderate
A1H1+Omalizumab (600) vs A1H1+Omalizumab (75)	1	No concerns	Low risk	No concerns	No concerns	No concerns	No concerns	High
A1H1+Omalizumab (600) vs Placebo+A1H1	1	No concerns	Low risk	No concerns	No concerns	Some concerns	No concerns	Moderate
A1H1+Omalizumab (75) vs Placebo+A1H1	3	No concerns	Low risk	No concerns	No concerns	No concerns	No concerns	High
CsA 4mg/kg/day+A1H1 vs Placebo+A1H1	1	No concerns	Low risk	No concerns	No concerns	No concerns	No concerns	High
MTX 15mg/wk+A1H1 vs Placebo+A1H1	1	No concerns	Low risk	No concerns	No concerns	No concerns	No concerns	High
Montelukast 10mg+A1H1 vs Placebo+A1H1	2	No concerns	Low risk	No concerns	No concerns	No concerns	No concerns	High
				Indirect evidence				
A1H1+Omalizumab vs A1H1+Omalizumab (150)	--	No concerns	Low risk	No concerns	Some concerns	No concerns	No concerns	Moderate
A1H1+Omalizumab vs A1H1+Omalizumab (300)	--	No concerns	Low risk	No concerns	No concerns	No concerns	No concerns	High
A1H1+Omalizumab vs A1H1+Omalizumab (600)	--	No concerns	Low risk	No concerns	Some concerns	No concerns	No concerns	Moderate
A1H1+Omalizumab vs A1H1+Omalizumab (75)	--	No concerns	Low risk	No concerns	No concerns	Some concerns	No concerns	Moderate
A1H1+Omalizumab vs CsA 4mg/kg/day+A1H1	--	No concerns	Low risk	No concerns	No concerns	Some concerns	No concerns	Moderate
A1H1+Omalizumab vs MTX 15mg/wk+A1H1	--	No concerns	Low risk	No concerns	No concerns	No concerns	No concerns	High
A1H1+Omalizumab vs Montelukast 10mg+A1H1	--	No concerns	Low risk	No concerns	No concerns	No concerns	No concerns	High
A1H1+Omalizumab (150) vs A1H1+Omalizumab (600)	--	No concerns	Low risk	No concerns	No concerns	No concerns	No concerns	High
A1H1+Omalizumab (150) vs CsA 4mg/kg/day+A1H1	--	No concerns	Low risk	No concerns	No concerns	Some concerns	No concerns	Moderate
A1H1+Omalizumab (150) vs MTX 15mg/wk+A1H1	--	No concerns	Low risk	No concerns	No concerns	No concerns	No concerns	High
A1H1+Omalizumab (150) vs Montelukast 10mg+A1H1	--	No concerns	Low risk	No concerns	No concerns	No concerns	No concerns	High
A1H1+Omalizumab (300) vs CsA 4mg/kg/day+A1H1	--	No concerns	Low risk	No concerns	No concerns	No concerns	No concerns	High
A1H1+Omalizumab (300) vs MTX 15mg/wk+A1H1	--	No concerns	Low risk	No concerns	No concerns	No concerns	No concerns	High
A1H1+Omalizumab (300) vs Montelukast 10mg+A1H1	--	No concerns	Low risk	No concerns	No concerns	No concerns	No concerns	High
A1H1+Omalizumab (600) vs CsA 4mg/kg/day+A1H1	--	No concerns	Low risk	No concerns	No concerns	Some concerns	No concerns	Moderate
A1H1+Omalizumab (600) vs MTX 15mg/wk+A1H1	--	No concerns	Low risk	No concerns	No concerns	No concerns	No concerns	High
A1H1+Omalizumab (600) vs Montelukast 10mg+A1H1	--	No concerns	Low risk	No concerns	Some concerns	No concerns	No concerns	Moderate
A1H1+Omalizumab (75) vs CsA 4mg/kg/day+A1H1	--	No concerns	Low risk	No concerns	Some concerns	No concerns	No concerns	Moderate
A1H1+Omalizumab (75) vs MTX 15mg/wk+A1H1	--	No concerns	Low risk	No concerns	No concerns	No concerns	No concerns	High
A1H1+Omalizumab (75) vs Montelukast 10mg+A1H1	--	No concerns	Low risk	No concerns	No concerns	No concerns	No concerns	High
CsA 4mg/kg/day+A1H1 vs MTX 15mg/wk+A1H1	--	No concerns	Low risk	No concerns	No concerns	No concerns	No concerns	High
CsA 4mg/kg/day+A1H1 vs Montelukast 10mg+A1H1	--	No concerns	Low risk	No concerns	No concerns	Some concerns	No concerns	Moderate
MTX 15mg/wk+A1H1 vs Montelukast 10mg+A1H1	--	No concerns	Low risk	No concerns	No concerns	No concerns	No concerns	High

(C)

Comparison	Number of Studies	Within-study bias	Reporting bias	Indirectness	Imprecision	Heterogeneity	Incoherence	Confidence rating
			Mixed evidence					
A1H1+Omalizumab vs Placebo+A1H1	1	No concerns	Low risk	No concerns	Major concerns	No concerns	No concerns	Low
A1H1+Omalizumab (150) vs A1H1+Omalizumab (300)	3	No concerns	Low risk	No concerns	Major concerns	No concerns	No concerns	Low
A1H1+Omalizumab (150) vs A1H1+Omalizumab (75)	2	No concerns	Low risk	No concerns	Major concerns	No concerns	No concerns	Low
A1H1+Omalizumab (150) vs Placebo+A1H1	3	No concerns	Low risk	No concerns	Major concerns	No concerns	No concerns	Low
A1H1+Omalizumab (300) vs A1H1+Omalizumab (75)	2	No concerns	Low risk	No concerns	Major concerns	No concerns	No concerns	Low
A1H1+Omalizumab (300) vs Placebo+A1H1	4	No concerns	Low risk	No concerns	Major concerns	No concerns	No concerns	Low
A1H1+Omalizumab (75) vs Placebo+A1H1	2	No concerns	Low risk	No concerns	Major concerns	No concerns	No concerns	Low
CsA 4mg/kg/day+A1H1 vs Placebo+A1H1	1	No concerns	Low risk	No concerns	Major concerns	No concerns	No concerns	Low
HCQ+A1H1 vs Placebo+A1H1	1	Some concerns	Low risk	No concerns	Major concerns	No concerns	No concerns	Low
MTX 15mg/wk+A1H1 vs Placebo+A1H1	1	No concerns	Low risk	No concerns	Major concerns	No concerns	No concerns	Low
Montelukast 10mg+A1H1 vs Placebo+A1H1	1	Some concerns	Low risk	No concerns	Major concerns	No concerns	No concerns	Low
			Indirect evidence					
A1H1+Omalizumab vs A1H1+Omalizumab (150)	--	No concerns	Low risk	No concerns	Major concerns	No concerns	No concerns	Low
A1H1+Omalizumab vs A1H1+Omalizumab (300)	--	No concerns	Low risk	No concerns	Major concerns	No concerns	No concerns	Low
A1H1+Omalizumab vs A1H1+Omalizumab (75)	--	No concerns	Low risk	No concerns	Major concerns	No concerns	No concerns	Low
A1H1+Omalizumab vs CsA 4mg/kg/day+A1H1	--	No concerns	Low risk	No concerns	Major concerns	No concerns	No concerns	Low
A1H1+Omalizumab vs HCQ+A1H1	--	Some concerns	Low risk	No concerns	Major concerns	No concerns	No concerns	Low
A1H1+Omalizumab vs MTX 15mg/wk+A1H1	--	No concerns	Low risk	No concerns	Major concerns	No concerns	No concerns	Low
A1H1+Omalizumab vs Montelukast 10mg+A1H1	--	Some concerns	Low risk	No concerns	Major concerns	No concerns	No concerns	Low
A1H1+Omalizumab (150) vs CsA 4mg/kg/day+A1H1	--	No concerns	Low risk	No concerns	Major concerns	No concerns	No concerns	Low
A1H1+Omalizumab (150) vs HCQ+A1H1	--	No concerns	Low risk	No concerns	Major concerns	No concerns	No concerns	Low
A1H1+Omalizumab (150) vs MTX 15mg/wk+A1H1	--	No concerns	Low risk	No concerns	Major concerns	No concerns	No concerns	Low
A1H1+Omalizumab (150) vs Montelukast 10mg+A1H1	--	No concerns	Low risk	No concerns	Major concerns	No concerns	No concerns	Low
A1H1+Omalizumab (300) vs CsA 4mg/kg/day+A1H1	--	No concerns	Low risk	No concerns	Major concerns	No concerns	No concerns	Low
A1H1+Omalizumab (300) vs HCQ+A1H1	--	No concerns	Low risk	No concerns	Major concerns	No concerns	No concerns	Low
A1H1+Omalizumab (300) vs MTX 15mg/wk+A1H1	--	No concerns	Low risk	No concerns	Major concerns	No concerns	No concerns	Low
A1H1+Omalizumab (300) vs Montelukast 10mg+A1H1	--	No concerns	Low risk	No concerns	Major concerns	No concerns	No concerns	Low
A1H1+Omalizumab (75) vs CsA 4mg/kg/day+A1H1	--	No concerns	Low risk	No concerns	Major concerns	No concerns	No concerns	Low
A1H1+Omalizumab (75) vs HCQ+A1H1	--	No concerns	Low risk	No concerns	Major concerns	No concerns	No concerns	Low
A1H1+Omalizumab (75) vs MTX 15mg/wk+A1H1	--	No concerns	Low risk	No concerns	Major concerns	No concerns	No concerns	Low
A1H1+Omalizumab (75) vs Montelukast 10mg+A1H1	--	No concerns	Low risk	No concerns	Major concerns	No concerns	No concerns	Low
CsA 4mg/kg/day+A1H1 vs HCQ+A1H1	--	Some concerns	Low risk	No concerns	Major concerns	No concerns	No concerns	Low
CsA 4mg/kg/day+A1H1 vs MTX 15mg/wk+A1H1	--	No concerns	Low risk	No concerns	Major concerns	No concerns	No concerns	Low
CsA 4mg/kg/day+A1H1 vs Montelukast 10mg+A1H1	--	Some concerns	Low risk	No concerns	Major concerns	No concerns	No concerns	Low
HCQ+A1H1 vs MTX 15mg/wk+A1H1	--	Some concerns	Low risk	No concerns	Major concerns	No concerns	No concerns	Low
HCQ+A1H1 vs Montelukast 10mg+A1H1	--	Some concerns	Low risk	No concerns	Major concerns	No concerns	No concerns	Low
MTX 15mg/wk+A1H1 vs Montelukast 10mg+A1H1	--	Some concerns	Low risk	No concerns	Major concerns	No concerns	No concerns	Low

Figure S2. Overall assessment of imprecision, incoherence, and heterogeneity of indirect and direct comparisons between omalizumab and other immunosuppressants. A. Daily Life Quality Index (DLQI), B. weekly urticaria activity score (UAS7), C. adverse events. We set clinically important mean differences of 8.9 and 14.0 for the DLQI and UAS7, respectively. For the adverse event assessment, we set an odds ratio of 1.0 for the following analysis. The incoherence assessment of the DLQI: X^2 statistic: 4.169, p=0.244, the incoherence assessment of UAS7: X^2 statistic: 5.64, p=0.396, the incoherence assessment of adverse events: X^2 statistic: 0.172, p=0.982. A1H1, H1-antihistamine; CsA, cyclosporin; HCQ, hydroxychloroquine; MTX, methotrexate.

(A)

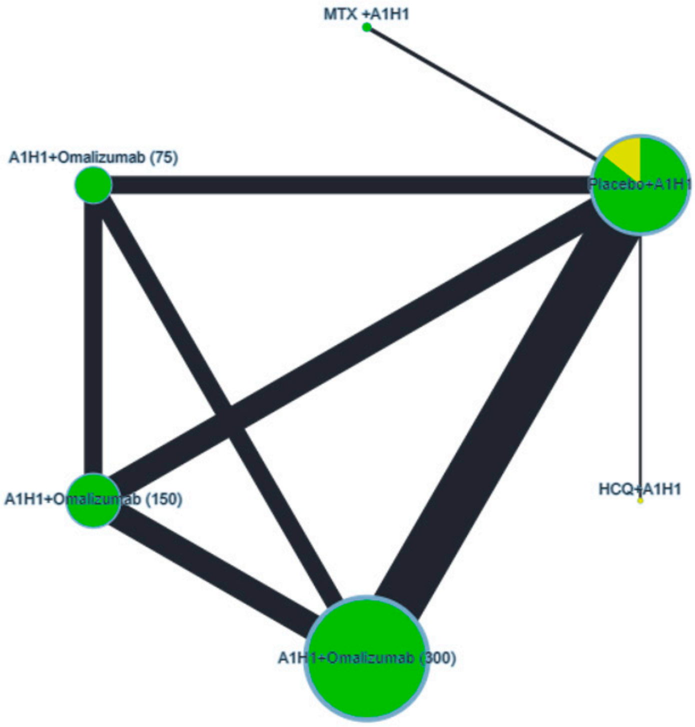
MTX +A1H1
A1H1+Omalizumab (75)
Placebo+A1H1
A1H1+Omalizumab (150)
HCQ+A1H1
A1H1+Omalizumab (300)

(B)

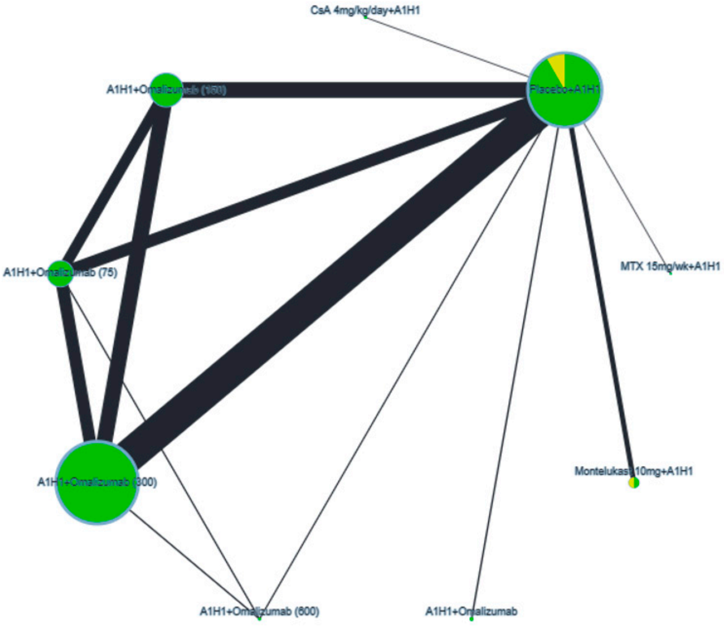
CsA 4mg/kg/day+A1H1
A1H1+Omalizumab (150)
Placebo+A1H1
A1H1+Omalizumab (75)
MTX 15mg/wk+A1H1
A1H1+Omalizumab (300)
Montelukast 10mg+A1H1
A1H1+Omalizumab (600)
A1H1+Omalizumab

(C)

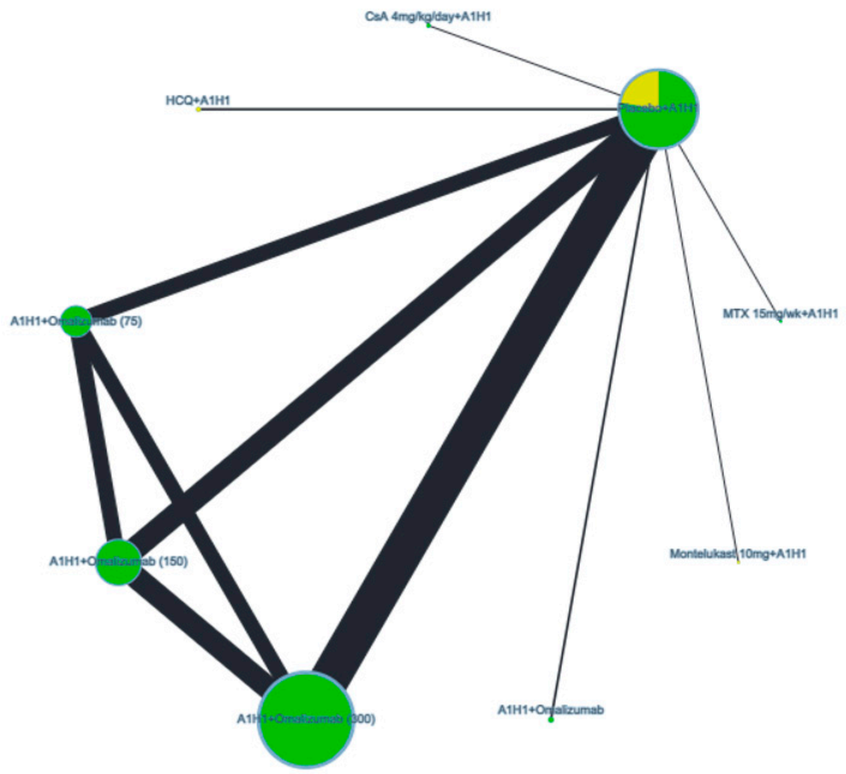

Figure S3. Network plot of all studies. A, Network plot of the Daily Life quality Index (DLQI) assessment, (B) network plot of the weekly urticaria activity score (UAS7) assessment, and (C) network plot of adverse effects. Nodes are weighted according to the number of samples included in related treatments. The widths of the edges are weighted according to the number of samples in each comparison. The color of the nodes demonstrate the risk of bias of included studies and are weighted according to the number of included studies; green means a low risk and yellow means some concerns. A1H1, H1-antihistamine; CsA, cyclosporin; HCQ, hydroxychloroquine; MTX, methotrexate.
